# Chronic Cannabidiol Administration Attenuates Skeletal Muscle De Novo Ceramide Synthesis Pathway and Related Metabolic Effects in a Rat Model of High-Fat Diet-Induced Obesity

**DOI:** 10.3390/biom10091241

**Published:** 2020-08-26

**Authors:** Patrycja Bielawiec, Ewa Harasim-Symbor, Karolina Konstantynowicz-Nowicka, Klaudia Sztolsztener, Adrian Chabowski

**Affiliations:** Department of Physiology, Medical University of Bialystok, 15-089 Bialystok, Poland; eharasim@umb.edu.pl (E.H.-S.); karolina.konstantynowicz@umb.edu.pl (K.K.-N.); klaudia.sztolsztener@umb.edu.pl (K.S.); adrian@umb.edu.pl (A.C.)

**Keywords:** cannabidiol, obesity, insulin resistance, ceramide, sphingolipids, glucose, insulin signaling

## Abstract

Numerous studies showed that sustained obesity results in accumulation of bioactive lipid derivatives in several tissues, including skeletal muscle, which further contributes to the development of metabolic disturbances and insulin resistance (IR). The latest data indicate that a potential factor regulating lipid and glucose metabolism is a phytocannabinoid—cannabidiol (CBD), a component of medical marijuana (*Cannabis*). Therefore, we aimed to investigate whether chronic CBD administration influences bioactive lipid content (e.g., ceramide (CER)), as well as glucose metabolism, in the red skeletal muscle (musculus gastrocnemius) with predominant oxidative metabolism. All experiments were conducted on an animal model of obesity, i.e., Wistar rats fed a high-fat diet (HFD) or standard rodent chow, and subsequently injected with CBD in a dose of 10 mg/kg or its solvent for two weeks. The sphingolipid content was assessed using high-performance liquid chromatography (HPLC), while, in order to determine insulin and glucose concentrations, immunoenzymatic and colorimetric methods were used. The protein expression from sphingolipid and insulin signaling pathways, as well as endocannabinoidome components, was evaluated by immunoblotting. Unexpectedly, our experimental model revealed that the significantly intensified intramuscular de novo CER synthesis pathway in the HFD group was attenuated by chronic CBD treatment. Additionally, due to CBD administration, the content of other sphingolipid derivatives, i.e., sphingosine-1-phosphate (S1P) was restored in the high-fat feeding state, which coincided with an improvement in skeletal muscle insulin signal transduction and glycogen recovery.

## 1. Introduction

Currently, obesity is a widespread medical condition reaching high rates in children and adults. Since 1975, the worldwide incidence of obesity increased almost threefold according to the World Health Organization [1]. The majority of obesity cases is the consequence of excessive food consumption and a sedentary lifestyle [2]. Sustained obesity disrupts metabolic processes and pathways, especially glucose and fatty acid (FA) metabolism, which is the background of lipotoxicity and insulin resistance (IR) [3], leading to the further development of metabolic syndrome (MetS) and type 2 diabetes mellitus (T2D) [4]. 

**Scheme 1:**
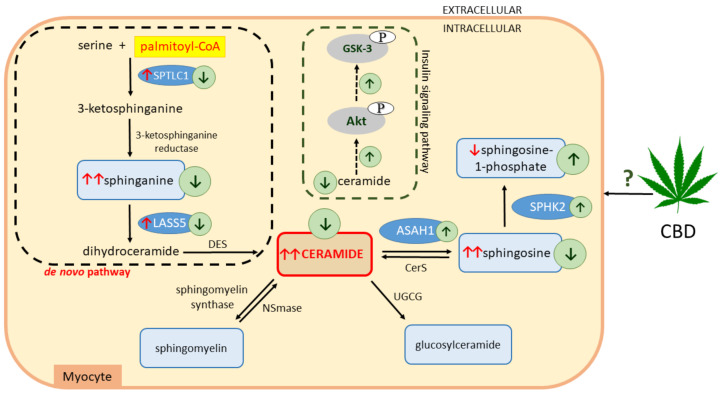
Effects of a high-fat diet (HFD) and chronic cannabidiol (CBD) administration on the sphingolipid metabolic pathway in rat myocytes. ↑, increase; ↓, decrease; red arrows indicate the effects of high-fat feeding; green arrows indicate the effects of CBD treatment; serine palmitoyltransferase, long chain base subunit 1 (SPTLC1); ceramide synthase 5 (LASS5); dihydroceramide desaturase (DES); neutral sphingomyelinase (NSmase); UDP-glucose ceramide glucosyltransferase (UGCG); ceramide synthase (CerS); acid ceramidase (ASAH1); sphingosine kinase 2 (SPHK2); phosphorylated protein kinase B (pAkt); phosphorylated glycogen synthase kinase 3 (pGSK-3).

An increased intake of fatty acids in a diet at an advanced stage of obesity progression results in adipocyte overload and the abnormal accumulation of bioactive lipid fractions in several tissues, including skeletal and cardiac muscle, as well as the liver [5,6]. Over the last several years, attention was paid to sphingolipids, the synthesis of which is increased during the overfeeding state (Scheme 1) [7]. Importantly, recent data clearly demonstrated that sphingolipids are not only structural components of the cells; they also act as signaling molecules participating in growth regulation, cell differentiation, apoptosis, and signal transduction [8]. It was shown that intramyocellular lipids, especially ceramide (CER), directly interfered with the insulin transduction signal pathway in the target tissues [5], which subsequently resulted in a deterioration of insulin-stimulated glucose uptake [9]. This seems to be of great importance since skeletal muscle, due to its mass, significantly contributes to the overall energy expenditure, for instance, by being responsible for nearly 80% of postprandial glucose uptake [9]. 

Currently, a new therapeutic approach is being sought for the prevention and treatment of obesity and coexisting disorders. The endocannabinoid system (ECS) was in the spotlight for several decades due to its well-established role in the regulation of appetite and energy expenditure [10,11]. It consists of cannabinoid receptors, CB1 and CB2, which are widespread throughout the body and located both in the central nervous system (CNS) and in the peripheral tissues (e.g., skeletal muscle, liver, adipose tissue) [12,13,14]. Cannabinoid receptors are sensitive to endogenous ligands (endocannabinoids (ECs)), mainly *N*-arachidonoylethanolamine (anandamide (AEA)) and 2-arachidonoylglycerol (2-AG), which are long-chain polyunsaturated fatty-acid derivatives [15]. The ECs, AEA and 2-AG, have their own metabolic routes, including enzymes responsible for their degradation: fatty acid amid hydrolase (FAAH) and monoacylglycerol lipase (MAGL), respectively [16]. Recently, new fatty-acid derivatives (more than 100), along with their corresponding molecular targets, were discovered, among which certain orphan G-protein-coupled receptors (GPCRs) like GPR55, transient receptor potential (TRP) channels like the TRP of vanilloid type-1 (TRPV1), and peroxisome proliferator activated receptors α and γ (PPARα and PPARγ) are present [17]. The above-mentioned components, cannabinoid and non-cannabinoid receptors, lipid mediators, and specific enzymes, based on recent findings, form the expanded ECS or endocannabinoidome (eCBome), which is an extension of the classic definition of the ECS [18]. 

Numerous studies showed that, during obesity, the ECS is overactivated; therefore, it emerges as a promising target in the treatment of obesity with a considerable physiological significance [19,20,21]. It was soon realized that cannabidiol (CBD) is a potential therapeutic agent on the grounds of its well-confirmed anti-inflammatory, anti-oxidative, anti-epileptic, anti-psychotic, and neuroprotective properties [22,23,24]. CBD is one of the most abundant and therapeutically relevant phytocannabinoids in the *Cannabis* plant, devoid of the psychoactive side effect [25]. So far, molecular targets involved in various therapeutic properties produced by CBD are not fully understood. Furthermore, few studies were performed in order to examine the effects of CBD with respect to obesity and its complications. Thus, the aim of the present study was to investigate whether chronic CBD administration affects the content of bioactive lipid species (e.g., CER or sphingosine-1-phosphate (S1P)), as well as insulin signal transduction, in red skeletal muscle (musculus gastrocnemius) of rats subjected to a high-fat diet (HFD) (Scheme 1). In our experimental model, we focused on the red skeletal muscle (consisting mainly of slow-twitch fibers) due to its predominant aerobic metabolism, in which a primary source of energy is based on the oxidation of glucose and FAs, as well as indicated insulin resistance [26].

## 2. Materials and Methods 

### 2.1. Animals and Study Design

Male Wistar rats (70–100 g) were purchased from the Center for Experimental Medicine of the Medical University of Bialystok, Poland. The animals were kept under controlled conditions (22 °C ± 2, 12-h/12-h light/dark cycle) with unlimited access to tap water and standard rodent chow (Labofeed B, Animal Feed Manufacturer “Morawski”, Kcynia, Poland). The study was approved by the Animal Ethics Committee in Olsztyn (No. 71/2018).

The animals were randomly assigned to four experimental groups after a period of acclimatization (seven days): (1) control group—rats fed a standard diet (kcal distribution: 12.4% of energy from fat, 57.1% from carbohydrates, and 30.5% protein), (2) CBD group—rats fed a standard diet and CBD-treated, (3) HFD group—rats fed a high-fat diet (kcal distribution: 60% of energy from fat, 20% from carbohydrates, and 20% protein), and (4) HFD + CBD group—rats fed a high-fat diet and CBD-treated. The total time course of feeding rats either standard chow or a high-fat diet lasted seven weeks, and each experimental group consisted of 10 rats. Starting from the fifth week, simultaneously with the respective diet, the rats received injections of CBD or its vehicle for the next two weeks of the experiment. Rats fed both a standard diet and an HFD were injected intraperitoneally (i.p.) with synthetic CBD (purity: ≥99%; THC Pharm GmbH, Frankfurt, Germany) in a dose of 10 mg/kg of body mass (3:1:16, ethanol, Tween-80, and 0.9% NaCl), and corresponding control and HFD groups received the vehicle once a day consecutively for 14 days. Twenty-four hours after the last dose of CBD or its solvent, rats from control groups, as well as HFD-fed groups, were anaesthetized by intraperitoneal injection of pentobarbital (80 mg/kg body mass). Muscle samples (red musculus gastrocnemius with oxidative metabolism) were collected, and visible fatty tissue was mechanically removed. Subsequently, the samples were immediately frozen using aluminum tongs precooled in liquid nitrogen and stored at −80 °C until further analyses. Blood samples were obtained through inferior vena cava puncture and collected into heparinized tubes and centrifuged; then, plasma was separated. 

### 2.2. Plasma Measurements

Plasma glucose and insulin concentrations were measured using a Glucose Colorimetric Assay Kit II (BioVision Inc., Milpitas, CA, USA) and Rat Insulin ELISA Kit (Mercodia AB, Uppsala, Sweden), respectively, following the manufacturer’s instructions. The intensity of colored product was measured in a hybrid multi-mode microplate reader (Synergy H1TM, BioTek Instruments, Winooski, VT, USA) and, for each measurement, calculated values were based on a separate standard curve. Additionally, the insulin sensitivity was assessed using the homeostasis model assessment of insulin resistance (HOMA-IR), where fasting plasma glucose (FPG) concentration was expressed in millimoles per liter and fasting plasma insulin was expressed in microunits per milliliter (HOMA-IR = (FPG × FPI)/22.5). 

### 2.3. Intramuscular Glycogen Analysis

The intramuscular glycogen content was determined using a colorimetric method (Glycogen Colorimetric Assay Kit II, BioVision Inc., Milpitas, CA, USA) according to the manufacturer’s protocol. Briefly, skeletal muscle samples were homogenized in double-distilled water; subsequently, tissues were boiled in order to inactivate enzymes and then centrifuged. Appropriate reagents were added to the collected supernatants and, after 30 minutes of incubation at room temperature. the absorbance of glycogen products was measured in a hybrid multi-mode microplate reader (Synergy H1TM, BioTek Instruments, Winooski, VT, USA). Calculated values were based on a standard curve, and glycogen concentration was expressed in micrograms per microliter.

### 2.4. Skeletal Muscle Lipid Analysis

The contents of ceramide (CER), sphinganine (SFA), sphingosine (SFO), sphinganine-1-phosphate (SFA1P), and sphingosine-1-phosphate (S1P) in the skeletal muscle samples were measured by the means of high-performance liquid chromatography (HPLC), as previously reported [27]. In brief, tissues were homogenized, and lipids were extracted by the addition of chloroform. The lipid extracts were transferred to a fresh tube with pre-added 40 pmol of *N*-palmitoyl-d-erythro-sphingosine (C17 base) as an internal standard. Afterward, the samples were washed with alkaline water to form deacylate ceramide. The obtained lipid residues released from ceramide were converted to their *o*-phthalaldehyde derivatives and analyzed using the HPLC system (PROSTAR; Varian Inc. (Palo Alto, CA, USA)) equipped with a fluorescence detector and C18 reversed-phase column (Varian Inc. OmniSpher 5, 4.6 × 150 mm).

### 2.5. Western Blotting

The total expression of proteins directly involved in sphingolipid and glucose metabolism, as well as components of the endocannabinoidome, was detected using a routine Western blotting procedure, as previously described [28]. Briefly, samples of the red skeletal muscle were homogenized in radioimmunoprecipitation assay (RIPA) buffer containing a cocktail of protease and phosphatase inhibitors (Roche Diagnostics GmbH, Mannheim, Germany). Then, the bicinchoninic acid method (BCA), with bovine serum albumin (BSA) as a standard, was used to ascertain protein concentration in the homogenates. After that, homogenates were diluted with Laemmli buffer, and the same amounts of protein (30 µg) were loaded onto CriterionTM TGX Stain-Free Precast Gels (Bio-Rad, Hercules, CA, USA). Subsequently, muscle homogenates were separated during electrophoresis and transferred onto nitrocellulose membranes. After blocking in Tris-buffered saline with Tween-20 (TBST) with 5% non-fat dry milk or BSA, the membranes were incubated overnight with selected primary antibodies: insulin receptor substrate 1 (IRS-1, 1:1000; Cell Signaling Technology, Danvers, MA, USA), phosphorylated insulin receptor substrate 1 (pIRS1 (Ser302), 1:1000; Cell Signaling), protein kinase B (Akt/PKB, 1:1000; Cell Signaling Technology), phosphorylated protein kinase B (pAkt/PKB (Ser473), 1:1000; Cell Signaling Technology), AS160 protein (AS160, 1:500; Cell Signaling Technology) phosphorylated AS160 protein (pAS160, 1:500; Cell Signaling Technology), glycogen synthase kinase 3 (GSK3, 1:500; Thermo Scientific, Rockford, IL, USA), phosphorylated glycogen synthase kinase 3 (pGSK3 (Ser9), 1:500; Thermo Scientific), glucose transporter 1 (GLUT1, 1:500; Santa Cruz Biotechnology, Inc., Dallas, TX, USA), glucose transporter 4 (GLUT4, 1:500; Santa Cruz Biotechnology, Inc., Dallas, TX, USA), pyruvate dehydrogenase (PDH, 1:5000; Abcam, Cambridge, UK), serine palmitoyltransferase, long chain base subunit 1 (SPTLC1, 1:500; Abcam), ceramide synthase 5 (LASS5, 1:500; Thermo Scientific), acid ceramidase (ASAH1, 1:500; Santa Cruz Biotechnology), sphingosine kinase 2 (SPHK2, 1:500; Sigma Aldrich, Saint Louis, MO, USA), cannabinoid receptor 1 (CB1, 1:500; Abcam), cannabinoid receptor 2 (CB2, 1:500; Abcam), transient receptor potential channel 1 (TRPV1, 1:500; Santa Cruz Biotechnology), and serotonin receptor (5-HT1A, 1:3000; Thermo Scientific). Next, nitrocellulose membranes were incubated with the corresponding secondary antibody conjugated with horseradish peroxidase (HRP) (Cell Signaling Technology). Thereafter, the protein bands were visualized using the appropriate substrate (Clarity Western ECL Substrate; Bio-Rad, Hercules, CA, USA), and obtained signals were quantified densitometrically with a ChemiDoc visualization system (Image Laboratory Software Version 6.0.1; Bio-Rad, Warsaw, Poland). The expression of selected target proteins was quantified using stain-free gels and the total protein normalization method (Bio-Rad). All data are expressed as the percentage of the control group based on six independent determinations. 

### 2.6. Statistical Analysis

All results are expressed as mean values ± SD. The data were subjected to the Shapiro–Wilk test and Bartlett’s test to assess the distribution of values and homogeneity of the variance. Statistical differences between groups were determined based on the results of one-way ANOVA followed by an appropriate post hoc test using GraphPad Prism version 7.0 for Windows (GraphPad Software, La Jolla, CA, USA). Results were considered to be statistically significant at *p* < 0.05.

## 3. Results

### 3.1. Effect of Chronic CBD Administration on Plasma Glucose and Insulin Concentrations, as Well as HOMA-IR, in Rats Subjected to Standard and High-Fat Diets

Our study demonstrated a pronounced decrease in plasma glucose level in the CBD group (−11.9%, *p* < 0.05; Table 1) compared to the control rats. Moreover, we noticed that both HFD-fed groups (untreated and treated with CBD) exhibited a significantly increased concentration of insulin (+89.9% and +45.2%, *p* < 0.05; Table 1, respectively) and considerably increased HOMA-IR index (+59.9% and +39.0%, *p* < 0.05; Table 1, respectively) in comparison with the control group. Importantly, we observed that two-week CBD treatment caused a substantial reduction in insulin concentration in the HFD group (−23.5%, *p* < 0.05; Table 1 vs. HFD group). Even though, chronic CBD administration decreased the HOMA-IR value in the HFD group compared to the corresponding untreated HFD group, the difference did not reach a significant level (−13.0%, *p* > 0.05; Table 1). 

### 3.2. Effect of Chronic CBD Administration on the Sphingolipid Pathway (Sphinganine, Sphinganine-1-Phosphate, Ceramide, Sphingosine, and Sphingosine-1-Phosphate) in Skeletal Muscle of Rats Subjected to Standard and High-Fat Diets

**Figure 1:**
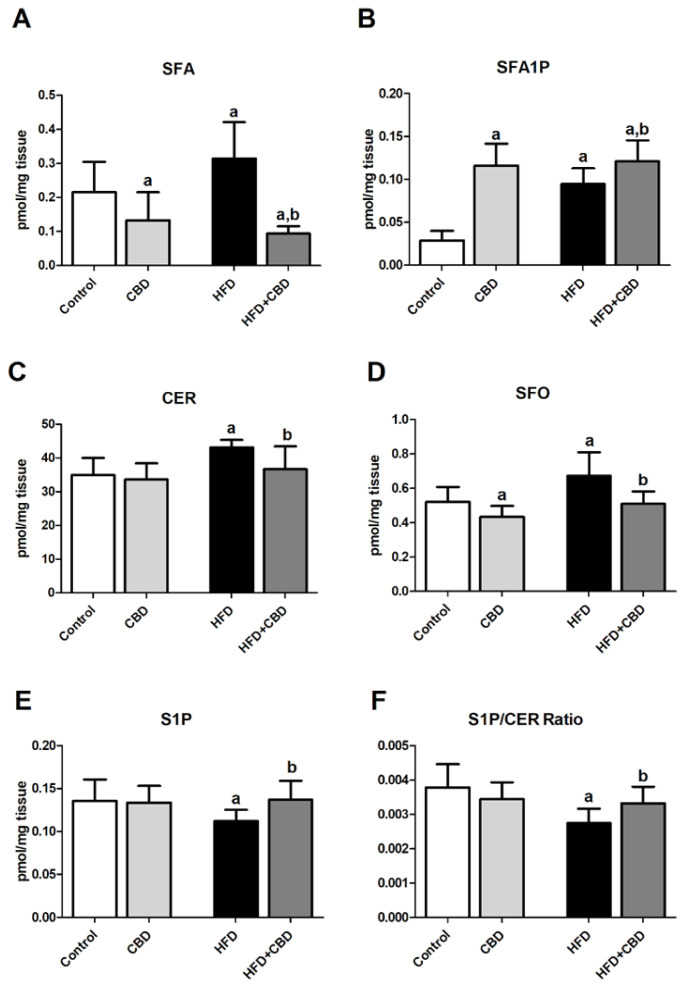
Intramuscular content of different components of sphingolipid pathway in rats after chronic cannabidiol (CBD) treatment, i.e., (**A**) sphinganine (SFA), (**B**) sphinganine-1-phosphate (SFA1P), (**C**) ceramide (CER), (**D**) sphingosine (SFO), (**E**) sphingosine-1-phosphate (S1P), and (**F**) sphingosine-1-phosphate/ceramide ratio (S1P/CER Ratio) in the red gastrocnemius muscle of rats fed a standard diet (control group) or high-fat diet (HFD). The data are expressed as mean values ± SD, *n* = 10 in each group. ^a^ *p* < 0.05 significant difference: control group vs. examined group; ^b^ *p* < 0.05 significant difference: HFD vs. HFD + CBD.

In the experimental model of HFD-induced obesity, we observed a significant intensification of the de novo ceramide synthesis pathway, which resulted in an elevation of intramuscular content of SFA (+21.2%, *p* < 0.05; Figure 1A), SFA1P (+231.1%, *p* < 0.05; Figure 1B), CER (+25.7%, *p* < 0.05; Figure 1C), and SFO (+14.8%, *p* < 0.05; Figure 1D) after the HFD course in comparison with the control group. As expected, chronic CBD administration to rats fed the high-fat diet substantially reduced the content of the above-mentioned components of the sphingolipid pathway, i.e., SFA (–72.9%, *p* < 0.05; Figure 1A), CER (−14.9%, *p* < 0.05; Figure 1C), and SFO (−24.3%, *p* < 0.05; Figure 1D), in the red gastrocnemius muscle compared to the HFD group alone. The only component of the sphingolipid pathway which was enhanced by two-week CBD treatment in rats fed either the standard chow or the high-fat diet in the red gastrocnemius muscle was SFA1P (+306.7% and +325.0%, *p* < 0.05; Figure 1B vs. control group, respectively). Concomitantly, compared to the control conditions, rats from the CBD group exhibited significantly reduced content of both SFA and SFO (−53.6% and −26.3%, *p* < 0.05; Figure 1A,D, respectively) with no change in CER and S1P levels (*p* > 0.05; Figure 1C,E, respectively). Interestingly, the intramuscular content of S1P was decreased in the lipid overload condition (−21.8%, *p* < 0.05; Figure 1E vs. control group) and subsequently elevated by CBD introduction in the same HFD group (+22.4%, *p* < 0.05; Figure 1E vs. HFD group). Similarly, the value of S1P/CER ratio was restored after CBD application in the high-fat diet group (−27.3%, *p* < 0.05; Figure 1F, HFD group vs. control group; +20.9%, *p* < 0.05; Figure 1F, HFD group vs. HFD + CBD group).

### 3.3. Effect of Long-Term CBD Administration on the Total Intramuscular Expression of Proteins Involved in the Sphingolipid Metabolism in Rats Fed Standard and High-Fat Diets

**Figure 2:**
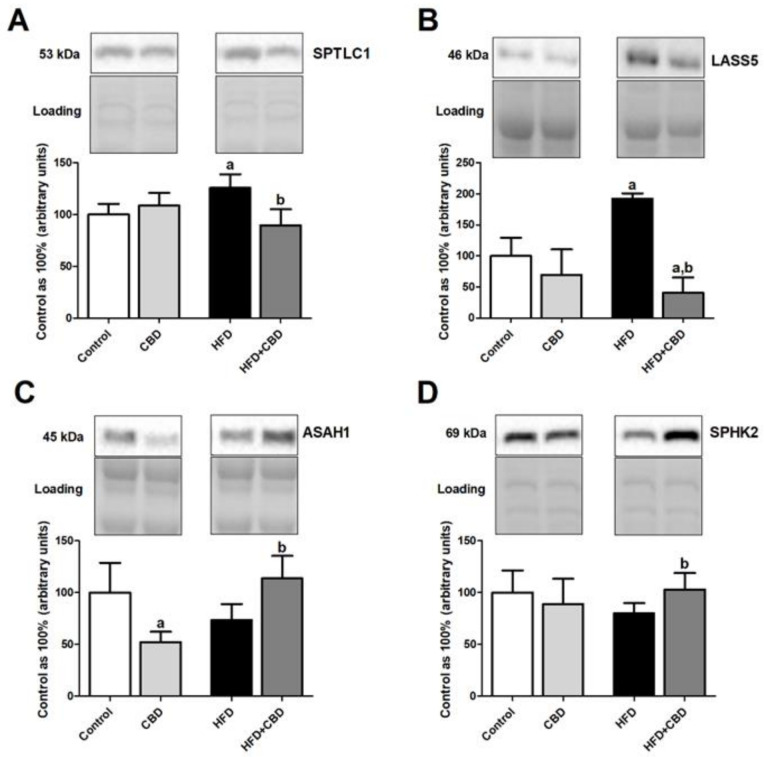
The total expression of proteins involved in the sphingolipid metabolism, e.g., (**A**) serine palmitoyltransferase, long chain base subunit 1 (SPTLC1), (**B**) ceramide synthase 5 (LASS5), (**C**) acid ceramidase (ASAH1), and (**D**) sphingosine kinase 2 (SPHK2), in the red gastrocnemius muscle in the control (standard diet) and high-fat diet (HFD) groups after chronic cannabidiol (CBD) treatment. The total expressions of the above proteins are presented as a percentage difference compared to the control group, which was set as 100%. The data are expressed as mean values ± SD, *n* = 6 in each group. ^a^ *p* < 0.05 significant difference: control group vs. examined group; ^b^ *p* < 0.05 significant difference: HFD vs. HFD + CBD.

Induction of obesity by high-fat diet feeding resulted in a significant increase in the total expression of SPTLC1 (+25.9%, *p* < 0.05; Figure 2A) in the red gastrocnemius muscle of the HFD group compared to the rats fed a standard chow, which was further declined by two-week CBD injections (−18.2%, *p* < 0.05; Figure 2A vs. HFD group). Similar effects compared to control conditions were observed in the high-fat diet group in regard to the total expression of LASS5 (+41.2%, *p* < 0.05; Figure 2B). Most importantly, the total intramuscular expression of this enzyme was considerably reduced in the chronic presence of CBD during the course of high fat feeding (−66.4%, *p* < 0.05; Figure 2B vs. control group and −76.2%, *p* < 0.05; Figure 2B vs. HFD group). Concomitantly, we did not notice any significant alternations in the total expression of ASAH1 and SPHK2 (*p* > 0.05; Figure 2C,D, respectively) in the red gastrocnemius muscle of rats subjected to an HFD compared to the control subjects. Interestingly, two-week CBD treatment had a more pronounced effect on the ASAH1 (+54.6%, *p* < 0.05; Figure 2C vs. HFD group) and SPHK2 expression (+28.7%, *p* < 0.05; Figure 2D vs. HFD group) in the red skeletal muscle during high-fat feeding. Moreover, during feeding rats with standard chow, CBD treatment had just the opposite effect of lowering ASAH1 expression (−47.6%, *p* < 0.05; Figure 2C) compared to the control group. 

### 3.4. Effect of Chronic CBD Administration on the Total Expression and Phosphorylation of Insulin Pathway Proteins, Glucose Transporters, and Glycogen Content in Skeletal Muscle of Rats Fed Standard and High-Fat Diets

**Figure 3:**
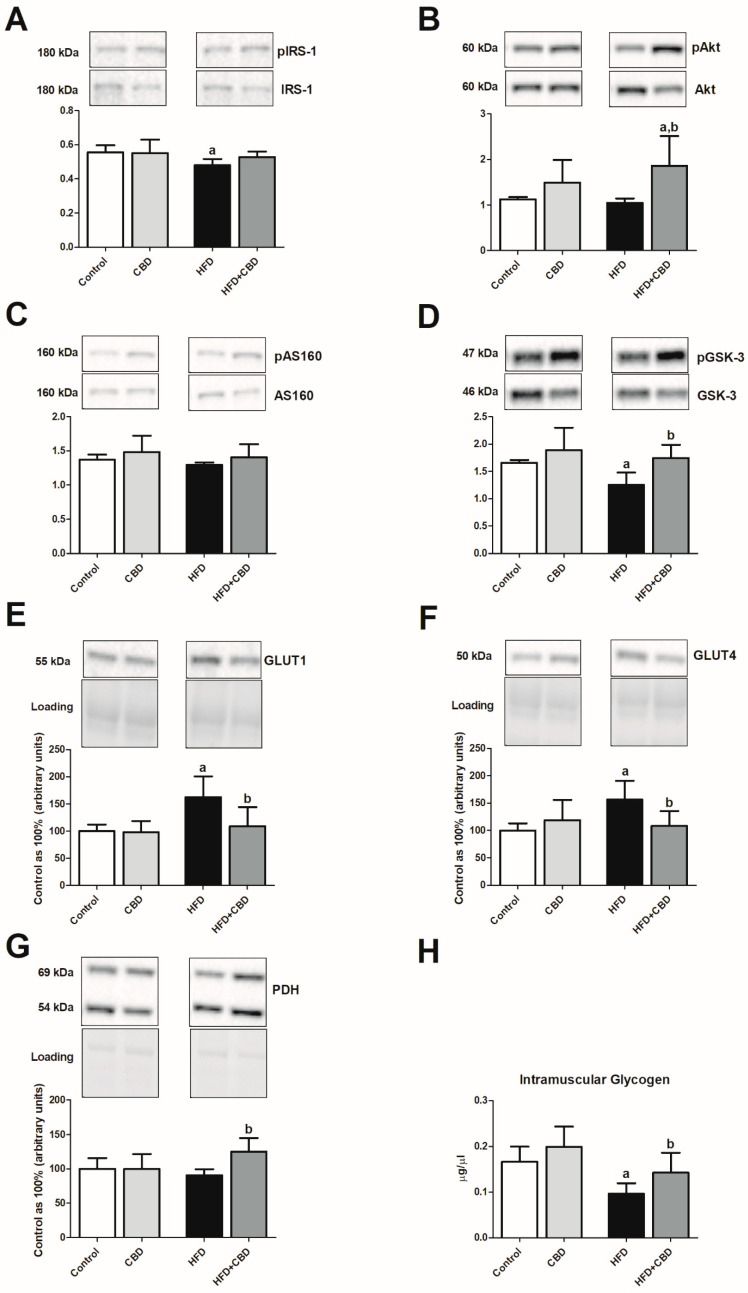
The ratio of total expression of phosphorylated and unphosphorylated proteins involved in insulin signaling pathway, e.g., (**A**) phosphorylated insulin receptor substrate 1/insulin receptor substrate 1 (pIRS-1/IRS-1), (**B**) phosphorylated protein kinase B/protein kinase B (pAkt/Akt), (**C**) phosphorylated AS160 protein/AS160 protein (pAS160/AS160), and (**D**) phosphorylated glycogen synthase kinase 3/glycogen synthase kinase 3 (pGSK-3/GSK-3), as well as total expression of (**E**) glucose transporter 1 (GLUT1), (**F**) glucose transporter 4 (GLUT4), (**G**) pyruvate dehydrogenase (PDH), and (**H**) glycogen content in the red gastrocnemius muscle in the control (standard diet) and high-fat diet (HFD) groups after chronic cannabidiol (CBD) treatment. The total expressions of the above proteins are presented as a percentage difference compared to the control group, which was set as 100%. The data are expressed as mean values ± SD, *n* = 6 in each group. ^a^ *p* < 0.05 significant difference: control group vs. examined group; ^b^ *p* < 0.05 significant difference: HFD vs. HFD + CBD.

In the skeletal muscle, we observed that rats fed an HFD showed a substantial decrease in the phosphorylation of proteins involved in insulin signaling pathway, i.e., IRS-1 (−13.8%, *p* < 0.05; Figure 3A, Figure S1A) and GSK-3 (−24.1%, *p* < 0.05; Figure 3D, Figure S1) in comparison with the control rats fed a standard diet. Concomitantly, we noticed a substantial elevation in the total muscular expression of GLUT1 and GLUT4 (+62.9% and +56.4%, *p* < 0.05; Figure 3E,F, respectively) with a parallel decrease in the content of glycogen in the red gastrocnemius muscle (−43.3%, *p* < 0.05; Figure 3H) in the HFD group compared to the control group. On the other hand, chronic CBD treatment resulted in a significant restoration in intramuscular phosphorylation of Akt (Ser-473) (+59.5%, *p* < 0.05; Figure 3B vs. HFD group) and GSK-3 (+38.4%, *p* < 0.05; Figure 3D vs. HFD group) compared to HFD alone. Concomitantly, CBD administration to animals being on an HFD resulted in a pronounced reduction of the total expression of both GLUT1 and GLUT4 (−32.9% and −30.8%, *p* < 0.05; Figure 3E,F, respectively) together with restoration of intramuscular glycogen pool (+56.4%, *p* < 0.05; Figure 3H) in comparison with HFD group. The above-mentioned effects of prolonged CBD treatment in high-fat diet rats were completed by markedly elevated total PDH expression (+37.9%, *p* < 0.05; Figure 3G vs. HFD group). 

### 3.5. Effect of Chronic CBD Administration on the Total Intramuscular Protein Expression of Endocannabinoid System Components in Rats Subjected to Standard and High-Fat Diets

**Figure 4:**
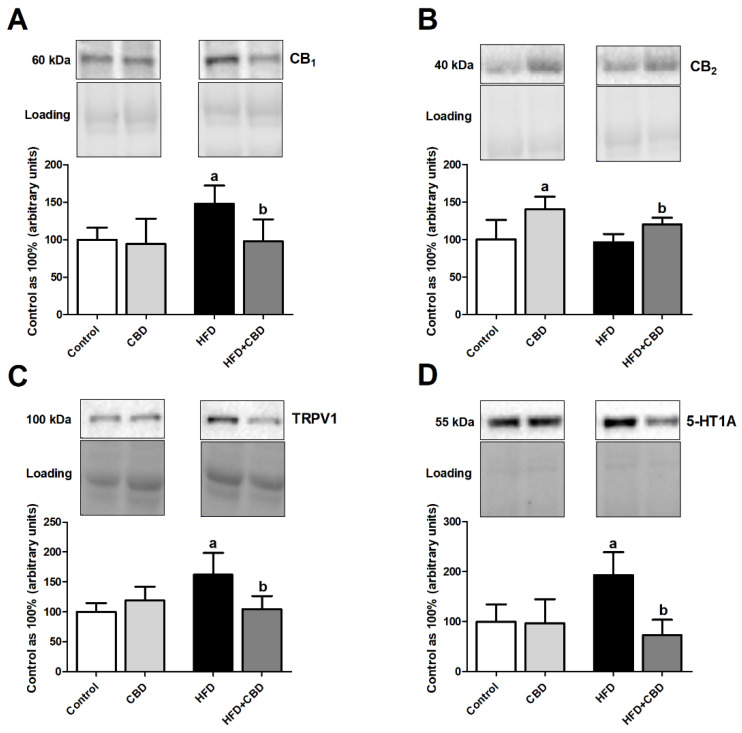
The total expression of (**A**) cannabinoid receptor 1 (CB_1_), (**B**) cannabinoid receptor 2 (CB_2_), (**C**) transient receptor potential channel 1 (TRPV1), and (**D**) serotonin receptor (5-HT1A) in red gastrocnemius muscle in the control (standard diet) and high-fat diet (HFD) groups after chronic cannabidiol (CBD) treatment. The total expressions of the above proteins are presented as a percentage difference compared to the control group, which was set as 100%. The data are expressed as mean values ± SD, n = 6 in each group. a *p* < 0.05 significant difference: control group vs. examined group; b p < 0.05 significant difference: HFD vs. HFD + CBD.

Our experiment demonstrated that the HFD group presented significantly elevated total expression of eCBome receptors, i.e., CB_1_ (+47.9%, *p* < 0.05; Figure 4A), TRPV1 (+61.9%, *p* < 0.05; Figure 4C), and 5-HT1A (+93.3%, *p* < 0.05; Figure 4D) in comparison with the control group fed standard chow. Unexpectedly, the total intramuscular expressions of the above-mentioned receptors in the lipid overload conditions and CBD presence were considerably decreased (CB_1_: −33.9%, *p* < 0.05; Figure 4A; TRPV1: −35.4%, *p* < 0.05; Figure 4B; 5-HT1A: −62.2%, *p* < 0.05; Figure 4D) compared to the rats subjected only to an HFD. Concomitantly, we did observe an increase in the total muscular expression of CB_2_ only in the case of animals fed a standard and high-fat chow and being injected with CBD (+40.6%, *p* < 0.05; Figure 4B vs. control group and +43.5%, *p* < 0.05; Figure 4B vs. HFD group).

## 4. Discussion

CBD, a non-psychotropic constituent of marijuana, exerts potentially beneficial pharmacological effects for obesity treatment. Therefore, according to our knowledge, for the first time, we examined, in a rat model of HFD-induced obesity and related whole-body insulin resistance, the impact of CBD on sphingolipids and glucose metabolism in the red skeletal muscle. It is important to note that the present study revealed the link between ceramide and other sphingolipid derivatives, ECS, and insulin signal transduction. 

As we mentioned, one of our aims was to determine the effect of CBD on the intramuscular content of selected sphingolipids (e.g., SFA, CER, S1P) in a model of HFD-induced obesity. During obesity, adipocytes are overloaded, which results in the accumulation of bioactive lipids in excessive amounts in several tissues, such as skeletal and cardiac muscle [29]. This phenomenon is known as lipotoxicity and contributes to the development of insulin resistance; however, the exact mechanism is not yet well characterized [30,31]. Accumulation of lipid intermediates, including CER, can directly interfere with the insulin signaling pathway [29]. In particular, ceramides impair insulin signal transduction by activating protein kinase C λ/ζ (PKC λ/ζ), implicated in the dephosphorylation and reduction in protein kinase B (Akt/PKB) activity, as well as stimulation of IκB kinase (IKK) and c-Jun N-terminal kinase (JNK), which attenuates insulin receptor substrate 1 (IRS-1) phosphorylation [32,33]. Importantly, our results demonstrated that CBD can be effective in ameliorating lipotoxicity and related insulin resistance due to observed alternations in the content of several sphingolipids. The current experiment showed markedly decreased SFA content after CBD treatment, thereby demonstrating a decline in the first step of the de novo pathway of ceramide synthesis, which was shown to be intensified during obesity [26]. Additionally, the above changes are in line with a decrease in SPTLC1 and LASS5 expressions (an enzymes involved in the de novo ceramide synthesis, Scheme 1) after CBD administration in rats fed an HFD [32]. This favorable effect of CBD action was manifested primarily by pronounced reduction in the intramyocellular CER content. Furthermore, ceramide derivatives, such as SFO and S1P, can also influence cellular survival, growth, and various functions and, thus, they may be involved in metabolic disorders [34]. SFO is reported as a proapoptotic molecule, whereas conflicting reports regarding S1P function in skeletal muscle can be found [35,36]. Several studies indicate that an increase in the S1P formation contributes to the IR [37]. However, a growing body of evidence described that S1P has just the opposite effects to CER and promotes cell proliferation and survival [38]. The current study showed a significant increase in intramyocellular SFO content in rats fed an HFD, whereas CBD substantially reduced its amount in favor of enhancing S1P content. This, in turn, resulted in an elevation of S1P/CER ratio and emphasized an improvement in sphingolipid rheostat imbalance due to IR [38]. The lower value of S1P/CER ratio observed in the HFD group may be associated with impaired insulin signal transduction, as well as enhanced cellular apoptotic processes. Furthermore, we observed changes in the total intramuscular expression of enzymes involved in the conversion of CER to its derivatives. Chronic CBD treatment of rats subjected to the fatty acid oversupply caused a considerable elevation in the total ASAH1 expression (conversion of ceramide to sphingosine; Scheme 1) and simultaneously reduced its expression in the standard diet group. These data confirmed that CBD also diminished accumulation of proapoptotic sphingolipids in rats fed a standard diet. Nevertheless, after CBD administration, we noticed an increase in the total SPHK2 expression, which is a kinase responsible for the maintenance of a balance between proapoptotic and proliferative precursors [38]. Moreover, Bruce et al. [39] demonstrated that SPHK1 overexpression prevents intramuscular ceramide accumulation by promoting its conversion into S1P and, thus, attenuates insulin resistance. Therefore, targeting enzymes involved in the maintaining an equilibrium between CER and S1P levels may be a beneficial strategy for improving muscle insulin sensitivity. Hence, it should be underlined that, in our research, we provide evidence for promising effects of CBD in regard to sphingolipid metabolism in the condition of lipid oversupply.

Moreover, our data showed that feeding rats an HFD attenuated whole-body insulin sensitivity since, after seven weeks of the experiment, we observed an increase in the concentration of insulin content. The occurrence of IR after a high-fat feeding was confirmed by the elevation in the HOMA-IR value, which is consistent with other researchers’ results [40,41,42]. In a number of studies, it was shown that increased fatty acid supply in a diet directly interferes with intracellular insulin signal pathways, leading to disturbances in whole-body glucose metabolism, together with a reduction of glycogen synthesis in skeletal muscle, which is again in line with the results obtained in our research [30,43,44]. Even though the data presented herein indicated that the two-week time frame of CBD treatment is too short in order to substantially improve whole-body IR, it was revealed that CBD ameliorates the deteriorated intramuscular insulin pathway in rats subjected to HFD, mainly by enhancing the phosphorylation ratio of proteins involved in the downstream signaling of that hormone (i.e., Akt and GSK-3). This is in line with the recent research conducted by Fellous et al., who showed that CBD treatment (5 μM) of bone marrow mesenchymal stem cells (BM-MSCs) prevented the palmitate-induced insulin resistance by increasing *Glut4* messenger RNA (mRNA) expression with simultaneous full restoration of Akt activation and subsequent glucose uptake [45]. In parallel, as the consequence of chronic CBD administration, we observed restored glycogen depletion in the red gastrocnemius, which resulted from increased phosphorylation of Akt and further GSK-3 inhibition. Furthermore, it is well established that a high-fat feeding results in impaired translocation of glucose transporter 4 from the intramyocellular compartments to the plasma membrane in insulin sensitive tissues [46,47]. In our study, we noticed considerably elevated total expression of both GLUT4 and GLUT1 in rats after a seven-week course of HFD, whereas CBD administration substantially reduced their skeletal muscle expression in the same group of examined animals. We hypothesize that the increase in the total expression of glucose transporters in HFD group was a compensatory effect, since parallel glycogen depletion was observed. Importantly, the aforementioned increase in GLUT4 and GLUT1 expressions was attenuated by chronic CBD treatment in the HFD group, most probably as the consequence of reduced plasma insulin concentration and an improvement in its downstream signaling (increased pAkt/Akt and pGSK-3/GSK-3 ratios) in red gastrocnemius. Even though, we did not measure plasmalemmal expression of GLUT4 in our study, it seems that CBD through regulation of signaling proteins stimulated intramyocellular trafficking of the GLUT4 transporter, which resulted in restoration of intramuscular glycogen and elevated expression of oxidative enzymes (i.e., increased expression of PDH). Moreover, we did observe alternations in the value of phosphorylated to unphosphorylated signaling proteins ratios only in the case of pAkt/Akt and pGSK-3/GSK-3, presumably due to lack of a direct stimulation of isolated skeletal muscle strips by insulin in ex vivo conditions, which may be considered as a limitation of the study.

Previous studies showed that ECS is overactivated during obesity and, thus, it became a potential target of therapeutic interventions [17,20]. In order to determine the effect of CBD on the ECS in skeletal muscle of rats fed a standard chow and HFD, we examined the total expression of cannabinoid (CB_1_ and CB_2_) and non-cannabinoid receptors (TRPV1 and 5-HT1A). Our experiment showed that high-fat feeding resulted in a substantial elevation of the total CB_1_ expression in a rat’s skeletal muscle. These results are consistent with findings of other researchers and may be associated with increased levels of endocannabinoids during obesity, especially AEA, which is a partial agonist of the CB_1_ receptor [17,18]. Furthermore, evidence was recently provided that CB_1_ activation induced by an HFD suppresses the insulin-dependent phosphorylation of Akt through IRS-1 phosphorylation at Ser-307, thereby mediating the emergence of insulin resistance [48]. Furthermore, Trillou et al. demonstrated that CB_1_^–/–^ mice are resistant to HFD-induced obesity [49]. Importantly, we noted that CBD treatment significantly decreased the expression of CB_1_ in the red gastrocnemius of rats subjected to an HFD. Our data are also in line with those obtained by Laprairie et al., since they demonstrated that CBD is a negative allosteric modulator of the CB_1_ receptor [50]. On the other hand, in the case of the CBD effect on CB_2_ receptors, several studies showed contradictory data, describing its activity as an agonist or inverse agonist of these receptors [51,52]. Our research reported that CBD significantly increased total CB_2_ expression in both control and high-fat diet-fed animals. The aforementioned alternations in the expression of cannabinoid receptors in skeletal muscle of high-fat diet-fed rats are in agreement with previous findings describing a positive relationship between CB_1_ receptors and oxidative stress [53] and an opposite effect in the case of CB_2_ receptors [53]. This should be underlined owing to the fact that obesity and related metabolic disturbances coincide with the promotion of oxidative stress. On the contrary, recent evidence emerged that, due to the low affinity of CBD for CB_1_ and CB_2_ receptors, it induces its effects primarily through other molecular targets, including TRPV1 channels and 5-HT1A receptors. The exact role of TRPV1 and 5-HT1A in obesity is not yet characterized, and additional research is needed to understand their molecular mechanism of action, as well as in the course of insulin resistance. In the current study, we showed elevated intramuscular TRPV1 and 5-HT1A expressions in HFD fed rats. We hypothesize that these changes may be associated with an increased level of endocannabinoids during obesity, in particular AEA, which is an agonist of those receptors [54]. Noteworthy, our research revealed that CBD administration to HFD-subjected animals resulted in a significant reduction in the expression of these receptors, indicating that CBD interferes with their activation. Such a conclusion arises since it is confirmed that CBD exhibits agonistic activity on the TRPV1 and 5-HT1A receptors [55,56]. Interestingly, recent research demonstrated that CBD, mostly via TRPV1 activation, enhanced murine C2C12 myoblast differentiation, together with inflammation reduction and autophagy restoration in in vivo conditions, which supported our notion concerning the protective role of CBD in the skeletal muscle [57]. Taken altogether, the data presented herein support the hypothesis that the ECS is involved in the development of metabolic disorders including insulin resistance. Moreover, this finding raises the possibility that CBD may be a useful tool in the treatment of obesity and its comorbidities by acting on the ECS, not only on receptors, but also on ligands and their metabolic routes.

## 5. Conclusions

In summary, our data provide new insight into the mechanism of cannabidiol action at the cellular level in skeletal muscle. We reported, for the first time, that chronic CBD treatment. on the one hand, prevented intramyocellular accumulation of CER and SFA, but, on the other hand, elevated S1P in FA oversupply in a diet. Moreover, we found that CBD improves downstream insulin signaling and the oxidative metabolism of glucose, while it restores glycogen depletion in myocytes during high-fat feeding. Furthermore, taking into consideration some limitations of the study, it seems that a two-week CBD treatment is too short to markedly diminish whole-body IR in obese subjects. Nevertheless, a two-week CBD treatment is enough to effectively inhibit the de novo ceramide synthesis pathway, thereby reducing lipotoxicity and provoking an insulin-sensitizing effect in the myocytes.

## Figures and Tables

**Table 1:** Plasma glucose and insulin levels, as well as homeostatic model assessment for insulin resistance (HOMA-IR), after chronic cannabidiol (CBD) administration in rats fed standard (control group) and high-fat diets (HFD). The data are expressed as mean values ± SD, *n* = 10 in each group. ^a^ *p* < 0.05 significant difference: control group vs. examined group; ^b^ *p* < 0.05 significant difference: HFD vs. HFD + CBD.

	Control	CBD	HFD	HFD + CBD
Glucose (mg/dL)	105 ± 8	93 ± 7 ^a^	94 ± 7	100 ± 6
Insulin (μg/mL)	0.65 ± 0.12	0.70 ± 0.18	1.24 ± 0.19 ^a^	0.95 ± 0.25 ^a,b^
HOMA-IR	3.84 ± 0.25	3.57 ± 0.77	6.46 ± 1.40 ^a^	5.62 ± 1.56 ^a^

## Supplementary Materials

The following are available online at https://www.mdpi.com/2218-273X/10/9/1241/s1: Figure S1. Representative Western blots showing the ratio of the total expression of phosphorylated and unphosphorylated proteins involved in the insulin signaling pathway.
