# Supplementary material for: Chronic Cannabidiol Administration Attenuates Skeletal Muscle De Novo Ceramide Synthesis Pathway and Related Metabolic Effects in a Rat Model of High-Fat Diet-Induced Obesity
Source: Biomolecules. 2020 Aug 26;10(9):1241. doi: 10.3390/biom10091241 (PMC7564398; doi:10.3390/biom10091241)
Supplement: Supplementary file 1 [file biomolecules-10-01241-s001.pdf]

Supplementary Materials

Supplementary Figure S1:

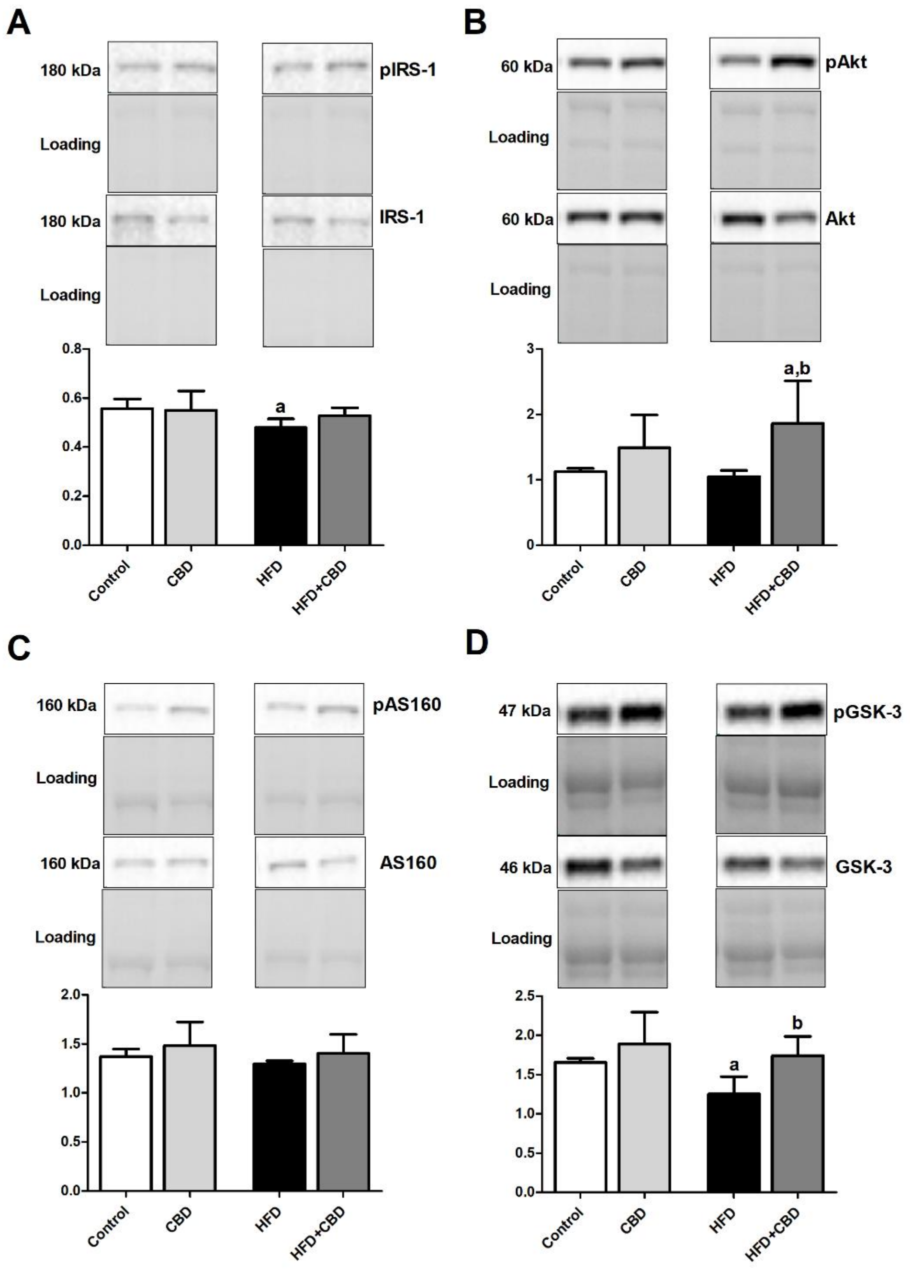

Figure S1: Representative Western blots showing the ratio of total expression of phosphorylated and unphosphorylated proteins involved in insulin signaling pathway e.g., (**A**) phosphorylated insulin receptor substrate 1/insulin receptor substrate 1 (pIRS-1/IRS-1), (**B**) phosphorylated protein kinase B/protein kinase B (pAkt/Akt), (**C**) phosphorylated AS160 protein/AS160 protein (pAS160/AS160) and (**D**) phosphorylated glycogen synthase kinase 3/glycogen synthase kinase 3 (pGSK-3/GSK-3) in the *red gastrocnemius* muscle in control and HFD groups after chronic CBD treatment. The total expressions of the above proteins were presented as a percentage difference compared to the control group, which was set as 100%. The data are expressed as mean values ± SD, $n = 6$ in each group. $^{a}p < 0.05$ significant difference: control group vs. examined group; $^{b}p < 0.05$ significant difference: HFD vs. HFD+CBD
